# Illness and femininity in Hilary Mantel’s *Giving Up the Ghost* (2003)

**DOI:** 10.1080/0950236X.2017.1371221

**Published:** 2017-09-08

**Authors:** Neil Vickers

**Affiliations:** Department of English Literature, King’s College London, London, UK

**Keywords:** Hilary Mantel, illness narrative, psychoanalysis, psychosomatics, phenomenology

## Abstract

This paper offers a reading of Hilary Mantel's memoir, *Giving Up The Ghost* (2003). The interest of the memoir derives from the fact that it provides an exceptionally rich picture of the impact of family life on a child's attitudes towards her own body. Mantel presents her bodily experiences as primitive, often unconscious, perceptions of the relationships within her family of origin. When she discovers new things about those relationships, she must register the change through her body in some way. Drawing on a range of concepts taken from psychoanalytic psychosomatics, I suggest that at the heart of the memoir is the author’s bafflement at the repeated and uncanny irruption of a conflict between her body as a somewhat autonomous signifying entity and the psychological strength she seeks and often finds through identifications with family members. I argue that this conflict overlapped with her acceptance of a female gender identity. The sustained nature of this conflict prevented her from establishing a metric of what I will call ‘psychosomatic normality’, with disastrous consequences when she began to suffer the symptoms of acute endometriosis. The memoir also shows the power of early life in determining how diseases are experienced subjectively, over time.

## Introduction

This is the second in a series of papers devoted to the body in life writing. Taking as my example Hilary Mantel’s memoir, *Giving Up the Ghost* (2003), I want to demonstrate how the relatively underused discipline of psychoanalytic psychosomatics might help scholars of life writing to understand the role of the body in the development of selfhood.^1^ The paper is part of a long-term project on illness narrative which seeks to explore how far illness memoirs (or autosomatographies, to use G. Thomas Couser’s much better term) by highly skilled writers might help us to understand what the experience of embodiment feels like, from the inside, in the West today.^2^ Growing up involves learning to cope with a wide range of bodily discomforts, even for healthy people. Such discomforts are part and parcel of what we might call psychosomatic normality. The point that literary autobiography illustrates so well is that bodily discomforts are never merely bodily just as psychological ones are never only mental. Both are compounds of the mental and the physical. Adapting to the rhythms of one’s psychosomatic reality is a productive process that enables the *self* to develop, not just the body; but this fact often escapes the attention of the healthy and the able-bodied because, as the physician-writer Eric Cassell so memorably put it, health is, among other things, ‘a mode of omnipotence’.^3^ I am particularly interested in autobiographies that lay bare the contribution that adapting to psychosomatic normality makes to selfhood. Life writing offers a valuable window onto the lived experience of psychosomatic normality and its vicissitudes.

To date, the most important significant large-scale theoretical attempt to take account of the physical sources of selfhood is that of Paul John Eakin.^4^ Taking his bearings from the work of the neuroscientist Antonio Damasio, Eakin is interested in the ways in which autobiographical memories survive as feeling-states to be elaborated in new contexts. Eakin’s implicit target was the poststructuralist claim that there is no necessary relationship between past and present selves. I want to broaden out Eakin’s model by considering the self as a psychophysical entity in relationship with other psychophysical entities. To fulfil this aim, I will appeal to concepts in psychoanalytic psychosomatics.

Psychoanalytic psychosomatics studies the effects of relational experiences on mind and body alike.^5^ In Mantel’s memoir, this relational dimension is especially prominent. She sets endometriosis and the devastating effect it has had on her life in the context of her exceptionally difficult childhood. I will suggest that her bodily experiences were shot through with primitive, often unconscious, perceptions of the relationships within her family of origin; whenever she discovered new things about those relationships, she found she had to register the change through her body in some way. She periodically defended herself from her mind by switching off from her body and from other people’s bodies, especially her mother’s (e.g. not noticing her mother was pregnant). These oscillations took place in the context of her growing acceptance of her female gender identity. There was, moreover, a special category of psychological experience which she connected with the supernatural; this changed her relationship with her body and which seemed to reinforce its tendency to debility. I will argue that supernatural experience was modelled on perceptions of her parents’ and step-parent’s relations with one another.

There is no unified psychoanalytic theory of what Winnicott memorably called the ‘psychosomatic partnership’.^6^ Consequently, the psychoanalytic ideas I shall draw upon come from an eclectic set of sources. I will appeal to Kristeva’s distinction between the semiotic somatic body and the symbolic somatic body but will add a Kleinian rider to it by insisting on the role of identification as a bulwark against the depredations of the semiotic.^7^ This is very marked in *Giving Up the Ghost*. I will also appeal to the idea of the *shared body schema*, first sketched out in detail by Maurice Merleau-Ponty, some version of which must underpin any version of psychoanalysis that sees the projection and introjection of bodily states as the most basic activity subtending all human life (i.e. all British variants). I will contend that the hardest struggle Mantel faced in early childhood was seeing her family of origin through her mother’s eyes. An intelligent, perceptive, imaginative child, who loved the rest of her family deeply, could only have been disturbed by what she saw. Developing a hypothesis by André Green concerning somatisation in general, I will suggest that a factor that could have led Mantel to live out so much of her psychic life through her body was she did not feel able to communicate her deepest experiences of family life to her parents or stepfather. Internal and external reality were poorly differentiated in her case, because of the reign of secrecy that governed family life.

The theories I shall advance will be set forth as plainly as I can, with only minimal recourse to psychoanalytic language, and they are intended *as speculations only*. For the avoidance of misunderstanding may I stress that in using the term ‘psychosomatics’ I disclaim any suggestion that Mantel’s endometriosis was in any sense *caused* by her sufferings as a child; but I do think childhood determined how she experienced the onset and development of that disease.

I have tried to frame these speculations in the light of contemporary biomedical research too. I worked for many years as a researcher in epidemiology. Mantel’s memoir contains a great deal of richly contextualised psychosocial material that illustrates and amplifies the findings of epidemiological studies on the long-term impact of early life stress.^8^ I highlight this aspect of her work because I think it is conceivable that in the future books like Mantel’s could come to play a more active role in medical research as generators and illustrators of hypotheses about the causes of illness and indeed of health. The interplay at the level of *lived experience* between the psychological and the physical is a primary focus for anthropologists of psychiatry such as Joan and Arthur Kleinman and Andrew J. Strathern and for theorists of narrative-based medicine; but it has received scant attention from literary scholars, even though literary autobiography is potentially an important source of information about that relationship.^9^

In the first part of this essay, I will describe the nature of Mantel’s mind–body interactions in early childhood, covering what she describes as the happiest part of her life, when she and her parents lived in her grandparents’ house. Young Hilary was especially fond of her grandfather, who doted on her.^10^ Strikingly, she believed she would turn into a boy on her fourth birthday or not long afterwards. In the second part I will consider the events that occurred following a holiday to Blackpool. It was then that she realised that her mother and father were unhappily married. This perception coincided with, and perhaps precipitated, a weakening of her hopes of turning into a boy. She became sickly. When she was six, the family moved to a new house, on a street named Brosscroft, not far from her grandparents’ home. Fairly quickly her mother installed her lover, Jack Mantel, in the new house. Mantel’s real father went on living in the house for around five years. During this period of intense domestic strife, Hilary was constantly ill with colics, pains, and fevers. The local GP nicknamed her ‘Little Miss Neverwell’. When she was seven, she had an experience in which she believed she could sense the presence of a supernatural creature, as high as a child of two, the embodiment of ‘some formless, borderless evil, that came to try to make me despair’ (p. 107). As a result of this experience, she was ‘never the same … I was always doomy afterwards’. She became ‘a graceless being, abandoned’ (p. 109). She passed the 11-plus exam in 1963 and the family, minus her father, moved to Cheshire. She never saw her father again. She attended an academically ambitious Catholic school in Cheshire, where she won the respect of the headmistress, Sister Mary Francis, ‘Top Nun’.^11^ The last section will consider the events surrounding her voluntary admission as a psychiatric inpatient for symptoms that included mysterious pains throughout her body which were thought to be hysterical at the time but which were almost certainly caused by endometriosis.

### Embodied symbolic knowledge

Julia Kristeva famously made a distinction between the *semiotic* somatic body, that is, the body which feels as though it can be acted upon by the world, and which takes the form of an awareness of sensation, and the *symbolic* somatic body, that is, the body through which a person acts upon the world so that she and it can be experienced as meaningful. The symbolic somatic body arises out of our growing confidence that brute somatic experience can be contemplated from a variety of viewpoints. The more we are able to withstand our bodily experience, the easier it becomes to dwell upon, play with, and reconfigure. For this reason, the semiotic somatic body is always on the way to becoming symbolic. Now, obviously, the extension of the semiotic into the symbolic realm is one of the signal achievements of infancy. In the sixth chapter of *Révolution du langage poétique* (1974), which contains her most extended discussion of the somatic and the symbolic, Kristeva relates the symbolic body to the child’s emergence into Lacan’s symbolic order. She says that the child who learns to speak is faced with ‘a dramatic confrontation between positing-separating-identifying and the semiotic *chora* [the body as we experience it from birth until the mirror stage which, according to Lacan, occurs when the child is aged between six and eighteen months]’.^12^ The semiotic body continues to exist in defiance of the symbolic as the repository of the pre-symbolic. Kristeva says that the differences between these two distinct states of the mind–body are especially significant in early childhood, when the symbolic body rises up out of the foundations of the somatic body for the first time.

Kristeva’s distinction captures something central to Hilary Mantel’s account of the pains of her childhood experience. *Giving Up the Ghost* is full of moments in which young Hilary submits to a bodily experience that threatens to overwhelm her. Sometimes it does overwhelm her and sometimes she manages to tolerate it and to sense its vast symbolic potential. I think that in the passages dealing with her childhood, Mantel is centrally preoccupied with the point at which the semiotic body metamorphoses into the symbolic body, or fails to do so, and that these experiences retained exemplary significance for her when she became ill. Here is an example of a successfully managed experience of this kind:

Evelyn’s house – the Aldous’s house – is darker than ours and has a more dumpling smell. Not being Catholics, they don’t have a piano, but as they are at the end of the common yard, they have a more tidy and well-arranged plot, with flower beds. Outside our house my granddad has grubbed out a bed for nasturtiums, and trained them up a wall. He calls them storshions … When I try to put names to their imperial colours, to the scarlet and striated amber, my chest seems dangerously to swell … (p. 43)

But just as often the proto-symbolic character of her experience is a source of tremendous anxiety to young Hilary. Near the beginning of the *Giving Up the Ghost*, Mantel talks of the ‘overwhelming sensory power’ of her early memories: ‘my early world was synaesthesic’, she writes, ‘and I am haunted by the ghosts of my own sense impressions, which re-emerge when I try to write, and shiver between the lines’ (pp. 24, 23). She recalls the pain of learning to walk from her grandmother’s house to the house next door, owned and inhabited by her great-aunt, Annie Connor. There is a raised bluish stone and ‘perhaps because it is the colour of a bruise, I will fall and howl’ (p. 29). To reach her destination, young Hilary had to pass a rusty iron ring: ‘Grandad says it is where they tied the monkey up but I don’t think they ever really had one; all the same, he lurks in my mind, a small grey monkey with piteous eyes and a long active tail’ (p. 28). When she masters the journey, she compares the sound of the piano in the house next door with the sound of her grandmother’s piano. The piano in her great-aunt’s house makes a ‘bronchial, damaged’ sound. Whenever it is played, young Hilary stands next to it ‘and feels the instrument resonating like a cat purring’ (p. 29). The purring in turn reminds her of a cat belonging to Mrs Clayton, a neighbour whose husband had recently died. Mrs Clayton was so distressed by her bereavement that she had to be admitted to a psychiatric hospital. The climactic memory in this sequence occurs when young Hilary sits on the stairs eating a marzipan sweet and wonders if she has swallowed a house-fly which can only mean that she will die soon. ‘There is another possibility, which I turn and examine in my brain: perhaps the tickling in my throat is the sweet itself … The fear of death turns slowly in my chest cavity, like a stewpot lazily bubbling’. ‘After a while’, Mantel writes,

I am walking about in the room again. My resolve to die completely alone has faltered. I suppose it will take an hour or so, or I might live till evening. My head is still hanging. What’s the matter? I am asked. I don’t feel I can say. My original intention was not to raise the alarm; also, I feel there is shame in such a death. I would rather just fall over, and that’s about it. I feel queasy now. Something is tugging at my attention. Perhaps it is a sense of absurdity. The dry rasping in my throat persists, but now I don’t know if it is the original obstruction lodged there, or the memory of it, the imprint, which is not going to fade from my breathing flesh. For many years the word ‘marzipan’ affects me with its deathly hiss, the buzz in its syllables, a sepulchral fizz. (p. 33)

The bluish flagstone does not merely foretell a fall; it is the *same* as a fall and the accompanying sense of physical pain. The absent monkey who has to be borne in mind stands for something worse than a fall. The child’s mind cannot compass what might have happened to this creature. The bronchial-sounding piano and the cat’s purring are frightening because they lead to Mrs Clayton’s grief which again is beyond her ken. The episode with the sweet that might be a fly involves an oscillation between the semiotic and a catastrophic version of the symbolic.

In her classic article on symbolism, Hanna Segal suggests that symbols should always be thought of as a ‘*three*-term relation, i.e. a relation between the thing symbolized, the thing functioning as a symbol, and a *person* for whom the one represents the other’.^13^ Young children use symbols as a means of throwing off anxiety. In the examples just given, the monkey, the piano and the cat symbolise mental pain (and possibly death itself) and in so doing serve to distance young Hilary from the reality of those things. But the things symbolised remain a penumbral presence. It is not uncommon for young children to be overwhelmed by symbolic significance: to find that the symbol makes the thing symbolised more alive. The marzipan sweet that turns into a fly and back into a sweet again is an example. It is more threatening than the ‘storshions’ because it brings forward a meaning that is attenuated in the other activities. I am suggesting that the mark of a successful symbol is that it lends itself to sublimation. The sweet is a better symbol of death than the fly because it pushes the idea of death further away.

One of the peculiarities of Mantel’s early childhood was the frequency with which her body was required to bear witness to meanings that she was not intended to understand. A beautifully described example takes place in Mantel’s grandmother’s house after the trip to Blackpool on which she registered her parents’ unhappiness. Her parents were playing chess.

A noise rips open the air. My parents raise their heads. It is a motorcycle, unsilenced, tearing open the afternoon, snarling down the street: 60 miles an hour. It rattles the windows: it is loud enough to wake babies, to frighten dogs. Then in an instant it has passed us, the noise is fading to a snarl; changing and dying in no time at all, to a long and melancholy drone, to a sigh. No one has spoken. But we have heard. Someone clears their throat: not me. They shift in their chairs. Their heads droop again. The racket, the roar, lasted for seconds, but the inner ear replays it and cannot help: winding away, with an afternote like vapour on the breeze, down the long and winding road.

I think I shall remember this. I shall remember this for ever; this dying note, the slanting light, their bent heads. It is a moment of pure self-consciousness, the foretaste of what is to come. I know, besides, that they are not looking at the chessboard; they are looking, covertly, at each other’s faces. (pp. 58–9)

It is the sublimation of the symbolic meaning of the motorcycle that is most impressive here. The tearing apart of the afternoon heralds the sundering of the parents’ marriage and the roar of the motorcycle arouses a moment of shared recognition even as it drowns it out. As children, we cannot know which parts of our lives are destined to develop and which parts are not. While retaining a great deal of her own privacy Mantel manages to evoke in her readers what psychoanalysts call ‘memories in feeling’ – events whose emotional tone we can recall without always having a clear idea of their content.^14^ She also manages to bring to life with particular delicacy the impact of not knowing certain specific things. Few if any children aged three or four understand their bodies or their parents’ relationship or the other relationships that structure their home life. Mantel’s achievement, I think, is to give the reader an idea of what *her* inability to see these things straight felt like. Coleridge (with Wordsworth in mind, of course) once defined genius as ‘the carrying on of the freshness and feelings of childhood into the powers of *manhood*’.^15^ By that yardstick, the early parts of *Giving Up the Ghost* are very ingenious indeed. Particularly impressive to me is the sustained rendering of the unthinkable weighing on the little girl’s mind in the form of physical experiences.

### Early identifications

Young Hilary seems to have found the thought of femininity to be unthinkable. Mantel does not tell her reader why she wanted to become a boy. All of the games remembered in the early part of the memoir involved imagining herself as male: a knight-errant or a priest. The person she seems to have most identified with during this period of her childhood was her grandfather. She notes that her grandfather had to be ‘knight and commander’ to all the women in the extended family. ‘His possessions are a billycan, a notebook and pencil, his guard’s hat and his guard’s lamp. It is my ambition to be a railway guard’ (p. 35). Her grandfather seems to have encouraged this identification, telling his granddaughter that he was unwilling to send her to nursery as she was too useful to him about the house.

Now identifications take us squarely into the realm of Kristeva’s category of the symbolic somatic body because they require the subject to experience her own agency. I want to suggest, however, that in addition to the love of her grandfather, Mantel’s early identifications were driven by a need to keep certain aspects of her home life out of focus. More specifically, Mantel used her symbolic body as a buffer between herself and the world of adult unhappiness.

Young Hilary was aware that her parents were very dissatisfied. They could not afford a home of their own, they could not get social housing because of anti-Catholic discrimination, and her in mother in particular seems to have been frustrated with her life.

Thwarted, unhappy, she stayed in the mill and earned, she said, a wage as good as a man’s. The work was hard and took a painful toll on immature muscle and bone. It would be many years before the effects showed; then, with energy to spare, she danced and sang through her evenings, in amateur shows and pantomimes. Cinderella was her favourite part. Her favourite scene: the Transformation. She asked herself, could she really be the child of her parents? (p. 50)

In later years, the mother complained to her daughter about her parents’ ‘narrow and unimaginative’ nature (p. 49). They had not allowed her to go to the grammar school where she would have thrived or entered her for a scholarship examination. They did not understand her wish to go to art college.

In one of the drafts of *Giving Up the Ghost* that appeared in the *London Review of Books* but which was not included in the book, Mantel describes the day she first met her stepfather, Jack, when she was four years old*.* She had gone with her mother to the primary school where her mother worked in order to borrow a typewriter. On the way home, Hilary turned around and noticed her mother walking with a man. After they go home, her mother says to everyone, ‘guess who I saw, Jack Mantel, Jack Mantel’.

It is an appealing tale, the tale of guess-who-I-met. Yet no one stays or lingers. No one pauses in their everyday routine, which includes running in and out of each other’s houses every few minutes. There seems, in general, to be a stony response to my mother’s news. I met Jack Mantel, Jack Mantel, she says. Her head is thrown back, her hair rippling to her shoulders, her voice trilling with laughter. She stands with one pretty calf advanced, one foot rocking in her high-heeled shoe. Guess who I met? No one answers. Her voice rises high and hangs itself on one of the vacant cuphooks on the shelves above my grandmother’s kitchen table.^16^

If this extract is authoritative, then Mantel’s mother’s relationship with Jack may have begun earlier than is apparent in the book. The memoir is more oblique. Mantel remarks that she lived in an environment where ‘The true nature of things was frequently hidden. No one would say plainly what was what: not if they could help it’ (p. 46). It is tempting to conjecture that in addition to the difficulties between her parents, there were also difficulties between her mother and her grandparents. These tensions, which simmered invisibly while the family lived in the grandparents’ house, quickly boiled over once they moved to Brosscroft.

The notion that she was fundamentally male also seems to have shielded young Hilary from knowing how unhappy her parents were. The link is first made when her parents take her for a holiday to Blackpool:

Standing on the pier at Blackpool, I look down at the inky waves swirling. Again, the noise of nature, deeply conversational, too quick to catch; again the rushing movement, blue, deep, and far below. I look up at my mother and father. They are standing close together, talking over my head. A thought comes to me, so swift and strange it feels like the first thought that I have ever had. It strikes with piercing intensity, like a needle in the eye. The thought is this: that I stop them being happy. I, me and only me. That my father will throw me down the rocks, down into the sea. That perhaps he will not do it, but some impulse in his heart thinks he ought. For what am I, but a disposable, replaceable child, and without me, they would have a chance in life. (p. 52)

I view this scene in terms of Hilary’s projective identification with her father: unconsciously, Hilary believed he saw her as the known-but-unknown Jack, and that he felt murderously angry with her as a consequence. But perhaps more important is the irruption of the semiotic body into the symbolic body.

The next thing I am in bed with a fever raging. My lungs are full to bursting. The water boils, frets, spumes. I am limp in the power of the current that tugs beneath the waves. To open my eyes I have to force off my eyelids the weight of water. I am trying to die and I am trying to live. I open my eyes and I see my mother looking down at me. She is sitting swiveled towards me, her anxious face peering down. She has made a fence of Mrs Scott’s dining chairs, their backs to my bed, and behind this barrier she sits, watching me. Her wrists, crossed, rest on the backs of the chairs; her lady’s hands droop. For a minute or two I swim up from under the water clawing … I feel myself taken by the current, tugged away. I am changed now. Not in that fever but in one of the series, one of those that follow it, my weight of hair is cut off. What remains is like feathers, I think, like fluff. I lose my baby fat. For another twenty-five years I will be frail. (pp. 52–54)

The symbolic body was a male body. When her parents’ misery forced itself upon her on Blackpool pier, her symbolic body was shattered and she was left with a semiotic body, acting upon her against her will. Her fantasy that she would turn into a boy lost a large share of its purpose, though that was not apparent to her at first. It seems clear that Hilary’s early difficulties with femininity were not based on denigration of the female body. Some of the most extraordinary passages of *Giving Up the Ghost* describe how overpowered she was by her mother’s beauty. They were, I suggest, rooted in a reluctance to consider the relationships in her family from her mother’s point of view. She was aware that she needed her mother and she suspected her mother wanted to abandon her. She saw how opposed her grandparents were to her mother’s wishes generally. And, once Jack was on the scene, she had to confront her mother’s hostility to her father and *vice versa*. If being a boy afforded her a measure of protection from these realities, why would she not seize it?

The conclusion of the scene is remarkable because it appears to hold out the promise of a reconstitution of a new symbolic body.

For a minute or two, I swim up from under the water: clawing. I think, how beautiful she [my mother] is. Her face frames a question. It is never spoken. My mother has brought her own bedlinen, from home, and below my hot cheek, chafing it, is a butterfly: spreading luxurious wings, embroidered on the pillow case by my mother’s own hand. I see it, recognise it, put out my hot fingers to fumble at its edges. If I am with this butterfly, I am not lost but found. But I can’t stay. I am too hot, too sick. I feel myself taken by the current, tugged away. (p. 53)

This primary instance of illness is fundamental. Blackpool supplies the symbolic language for every subsequent collapse in the memoir.^17^ It also describes a kind of ‘arc’ of her imperfect recoveries from each subsequent episode of illness: the irruption of the semiotic body into the symbolic, followed by the precarious establishment of a new symbolic body *based on identification with a different member of her family*.

### The shared female body schema

The embroidered butterfly is a symbol of her own female body and it is the occasion for one of the rare moments dealing with childhood in which Hilary appears to value her own femininity. In a previous paper, I suggested that one of the distinguishing marks of memoirs involving illness is the frequency with which the writer discovers unconscious somatic memories. In particular, the sick person finds that he or she shared a body schema with a person or persons who are important to them. Strikingly, we are just as likely to share a body schema with someone of the opposite sex as with someone of our own. This phenomenon has been studied by scholars in a wide variety of scientific and non-scientific fields. Vittorio Gallese, one of the discoverers of mirror neurons in macaque monkeys, has proposed that whenever humans look at someone performing an action, our motor system becomes active as if we ourselves were executing the action in question.^18^ A recent number of *Behavioural and Brain Sciences* was devoted to the theme of ‘Second-Person Neuroscience’ in support of this hypothesis.^19^ Gallese and his colleagues acknowledge the impact of research into infant development which infants as young as six hours appear to have a capacity to imitate those around them. Daniel N. Stern and Giannis Kugiumutzakis have described experiments in which a grown-up sticks out her tongue or opens her mouth at an infant and the infant does the same thing back.^20^ We know it is not a reflex because infants do not always do it; but they do it a great deal. The baby has to translate the experience of seeing someone into a proprioceptive impression which forms the basis of their imitation. They do not look at their own tongues or hands or whatever it is they are using to imitate the grown-up. They just do it. Seeing others’ actions give us a template for action. We do not consciously copy them; rather we discover that we have copied them through feeling and action. This is the most rudimentary form of intersubjectivity and it is rooted in intercorporeality. Philosophically, the father of these views is Husserl who emphasised that our capacity to share experiences with others turns on a process by which we simulate their experiences in our own bodies. Merleau-Ponty developed this claim by suggesting that at some level we are obliged to fuse our experience with that of other people.

In perceiving the other, my body and his are coupled, resulting in a sort of action which pairs them *[action à deux].* This conduct which I am able only to see, I live somehow from a distance. I make it mine; I recover *[reprendre]* it or comprehend it. Reciprocally I know that the gestures I make myself can be the objects of another’s intention. It is this transfer of my intentions to the other’s body and of his intentions to my own, my alienation of the other and his alienation of me, that makes possible the perception of others.^21^

I think this theory or one like it implicitly underpins Melanie Klein’s idea of projective identification for this last assumes that we can have, and at some level believe we do have, the same bodily experiences as other people, and that they can have and do have ours.^22^ The only additional claim I wish to make is that some shared body schemas become part of our long-term experiences of embodied selfhood and radically shape our sense of who we are. This can be seen very clearly in memoirs dealing with life-changing illness.

Now it is plain from the early parts of *Giving Up the Ghost* that if Hilary felt her body was coupled with anyone else’s, it was her grandfather’s. Under his tutelage, she could move about in the world endowed with his power. Even the meals they ate seemed to confirm their connection in her mind:

Grandad and I have special food, at different times from other people. When he comes off his shift he eats alone, tripe, rabbit, distinctive food that is for men. Around noon each day I take a lamb chop, and a slice of bread and butter. (p. 45)

The illnesses she suffered post-Blackpool entailed losing his body as a happy source of representation for her own. She continued to play boyish games though increasingly these now felt like play to her. If Merleau-Ponty’s account of the developmental significance of the shared body scheme is correct, it would have been impossible for Hilary to be at ease with her female body without an admired authoritative female figure near at hand whose bodily experience offered an attractive pattern for her own. The problem was that few things made Hilary more anxious than her mother’s body and its capacities. And yet precisely because her mother was such a source of anxiety she could not but dominate her daughter’s mind.

Hilary had to come to terms with being female in a context in which she felt extremely unwell. The illnesses described are well documented in the medical literature on childhood stress.^23^ Stress compromises the immune function, especially in early life. Fevers are commonplace, the result of an excessively active immune system. The normal cycle of hair growth ceases, leading to temporary hair loss (the phenomenon known as ‘telogen effluvium’). And the pains and fatigue mentioned could easily have been caused by excess cortisol in the body. The GP who nicknamed Hilary ‘Little Miss Neverwell’ appears to have been ignorant of the physiology of stress. But perhaps he was struck by the intense mutual involvement of mother and daughter during illness. Hilary feared her mother’s waywardness very much. Through illness, the mother appears to have identified with the daughter. Maybe it was a way of bestowing on her daughter the care of which she had felt deprived. At any rate the sick body was perhaps the first version of the shared *female* body schema.

Hilary’s emerging sense of her body as female appears to have turned on two related intuitions: that it was less substantial than the male body and more prone to illness. Arguably the most disturbing feature of her life after Blackpool was the degree to which illness and femininity became constitutive of one another.

I am only playing, inside the Indians tepee, and I know it. I have lost the warrior’s body I had before fever. My bullet-like presence, my solidity, has vanished. Ambiguity has thinned my bones, made me light and washed me out, made me speechless and made me blonde. I realise – and carry the dull knowledge inside me – that I am never going to be a boy now. I don’t exactly know why. I sense that things have slid too far, from some ideal starting point. (p. 57)

A body made thin and translucent by illness was implicitly female. Here, for instance, is her description of her life at the moment when her parents quarrelled over Jack moving in.

This is the worst time of my life: days of despair. I am on the pier at Blackpool, with the screaming gulls and the wind, looking down into the boiling sea. Words swirl over my head, words of loathing and contempt. A great hand lifts me; it is the hand of the law. And here is my punishment, coming now, coming now; I feel the rush of air against my face. The law picks me up into the wind, the law lets me go; I fall through space, and on the rocks my head smashes open like an egg. The sea drinks my yellow blood. (pp. 84–5)

When she was actually in Blackpool her worry was about being disposed of, violently. Now the focus is on her body disgorging its sickly sounding contents (yellow blood). From this point on, the memoir has a great deal to say about internal bodily spaces. The Maudsley psychoanalyst Henri Rey thought that the consciousness of internal bodily spaces was the defining feature of the sense of femininity.^24^ It is striking how often Mantel refers to the insides of her body in her account of her life in Brosscroft.

The memoir is quite brilliant on how these conflicts about her own body took shape around her relationship with her mother. As relations with the rest of the family worsened, Hilary oscillated between identifying with her mother and cutting off from her. Identification took four main forms: (1) imaginative play in which she directly imitated her mother^25^; (2) a deepening sense of herself as the child of parents who did not understand her (echoing her mother’s experience of *her* parents); (3) unconscious projective identification with her mother’s hidden life^26^; and (4) as she moved into adolescence, a desire to vindicate her mother’s thwarted academic ambitions. She also tried, with some success, to switch off from her body. She became a feared playground fighter at school (‘[my body] has no capabilities and no capacities, except to be in the way, to be where it’s not wanted’). She cut off from her senses and again, as if to confirm Merleau-Ponty’s notion of the use of a shared body schema, the point of cutting off from her senses was to blunt her sense of her mother’s body. For a time, she could not hear what her mother said to her, forgot the colour of her mother’s hair, and failed to notice when her mother was pregnant (p. 65).

The final acceptance of femininity seems to have been occurred for Hilary in the climactic episode of the chapter entitled ‘The Secret Garden’. Shortly before her First Communion, Hilary ‘carried a simple space for God inside me: a jagged space surrounded by light, a waiting space cut out of my solar plexus’. This space was invaded by the vision of the evil spirit which seems to have come to her not so much through her eyes as through her heaving stomach and her sense of touch:

My eyes are drawn to a spot beyond the yard, beyond its gate, a spot in the long garden … I can’t see anything, not exactly see: except the faintest movement, a ripple, a disturbance of the air. I can sense a spiral, a lazy buzzing swirl, like flies; but it is not flies. There is nothing to see. There is nothing to smell. There is nothing to hear. But its motion, its insolent shift, makes my stomach heave. I can sense – at the periphery, the limit of all my senses – the dimensions of the creature. It is as high as a child of two. Its depth is a foot, fifteen inches. The air stirs around it, invisibly. I am cold, and rinsed by nausea. I cannot move. I am shaking; as if pinned to the moment, I cannot wrench my gaze away. I am looking at a space occupied by nothing … Within the space of a thought, it is inside me, and has set up a sick resonance within my bones and in all the cavities of my body. (pp. 106–7)

After this vision, she was ‘never the same … I was always doomy afterwards’. She became ‘a graceless being, abandoned’ (p. 109).

I want to make two suggestions about this scene. First, this encounter with a supernatural being was based on a fantasy of impregnation by, and with, a creature the height of a child of two. At the time this scene took place, the older of her two brothers, Ian, was two years old.^27^ The scene is thus fundamentally one in which Hilary identifies with her mother’s body. It is about the psychological cost of protecting her mother from knowing how disturbed she (Hilary) had been by what her mother did to her family. More specifically, the intensely curious part of the little girl avoids communicating to her mother how damaging she finds adult sexuality by projecting all her own hatred (and the hatred she believes her mother’s sexuality contains) into her sibling. She is relating to her mother but the line of contact is distorted by the projection that turns her sibling into an evil spirit. If on Blackpool Pier she learned that no amount of thinking she was a boy could remedy her parents’ unhappiness, in the Secret Garden, she intuited that being female meant having a body that is either vulnerable to requisition by others (men, babies, supernatural entities) or that can draw others into its own vortex and that this peculiarity is based on their having distinctive internal bodily space.^28^ Again, the result of this intuition is the destruction of her (female) symbolic body as a source of happy representation. It is, perhaps, the moment in which the permanence of her link with her mother’s body became apparent to Hilary. It is as if, from this point, Hilary stopped expecting to feel well. As a result of this vision, she writes, she was ‘never the same … I was always doomy afterwards’. Elsewhere, Mantel has written movingly about the impact of chronic pain on her personality and intellect, beginning in childhood.^29^

My second observation is more speculative and I will explain it fully only in the next part of this essay. The religious terms in which she perceived her plight may have come from her Catholic grandmother. The tainted version of herself she was left with involved an identification with her grandmother’s image of her mother. Yet again, we find identification being used as a bulwark against the complete destruction of her symbolic body but it is a double identification with mother and grandmother, offering no clear resolution, not unlike her ‘trying to die and trying to live’ in Mrs Scott’s boarding house.

Perhaps I might make a general point about the meaning of the supernatural in *Giving Up the Ghost*. Many commentators have drawn attention to Mantel’s metaphoric use of the word ‘ghost’ in the memoir. She talks about the ‘ghosts’ of the children she never had and of the boy she never became as negative existences that continue to define her. This version of ghostliness will offend no one. In what is perhaps the best discussion of the topic, Alan Radley suggests that ‘Mantel’s ghosts are misunderstood if they are interpreted either as objects or as hallucinations. They are key mediators of the work that is her story’.^30^ Although I heartily concur with him on the last point, every reader of *Giving Up the Ghost* has to confront the fact that Mantel gives us ghosts in an objective, hallucinatory form and it is these which are most troublesome (including for this writer). I think that ghosts that caused havoc in Brosscroft enabled the little girl to register a point of view that dominated family life but which was seldom openly proclaimed. This point of view had to do with the passions and apprehensions generated by the adults’ unusual living arrangements. Believing in ghosts was a way of registering those passions without having to allude to them directly. This can be seen in the passage in which the word ghosts first makes its appearance in family life:

The dogs, who are no longer puppies, squeal with fear in the night. My mother comes down to them, shivering in her nightdress, and sees their hackles raised, their thin forms shrinking against the dawn light. One night, I hear my mother and Jack, discussing. I am lurking in the cold Glass Place, coming in from the lavatory. ‘Well,’ she says, coming in from the lavatory. ‘Well,’ she says, ‘so? So what do you think it is?’ Her voice rises, in an equal blend of challenge, fear and scorn. *Ghosts*?’ She has spoken my thoughts: which I thought were unspeakable. The hairs rise on the back of my neck. (pp. 95–6)

In an important interview with Eileen J. Pollard for this journal, Mantel remarked that she believes in ghosts ‘for practical purposes’.^31^ The ‘practical purposes’ in question were about getting hold of the emotional currents all around her and making as much sense of them as she could, without betraying their overwhelming power. It was almost inevitable that it should be bound up with sexuality.

### Endometriosis

In 1970, Mantel won a place to read Law at the London School of Economics but transferred to Sheffield the following year, to be near her boyfriend and future husband. There she reported ‘a pain which I could not explain; it seemed to wander about my body, nibbling here, stabbing there, flitting every time I tried to put my finger on it’ (p. 149). Many years later she established that these pains were almost certainly caused by endometriosis, a notoriously difficult condition to diagnose, especially in 1970, but as her doctors could find no physical cause for them at the time, they concluded that they were probably psychogenic. Her GP put her on tricyclic antidepressants; her pains continued. She was then sent to see a psychiatrist, Dr G. Dr G thought she was a hysteric. He said her ailments stemmed from the fact that she was a law student. The law, he told her, was too intellectually demanding a subject for a woman, especially one as conscientious as Mantel. He advised her to give up her studies and to get a job in a dress shop, like her mother (p. 170). (Her mother was in fact a fashion buyer for a major department store in Manchester.) Dr G prescribed Valium; but instead of tranquillising her, Mantel found that the drug made her furious: she wanted to burn down buildings. She admitted herself into the care of a psychiatric unit as a voluntary patient, to reduce the likelihood of committing arson. Dr G thought she was sliding rapidly into psychosis and he put her on antipsychotics to which she had an akathisic, psychotic, adverse reaction. It took several weeks for her doctors to see that her psychosis was iatrogenic. She resolved to endure her pains and to steer clear of psychiatrists forever. Her endometriosis worsened until she diagnosed it herself some eight years later by which time it was too late to save her womb.

Many of the first reviews of *Giving Up the Ghost* presented this episode as the most significant event in the whole memoir. ‘The more I said that I had a physical illness, the more they said I had a mental illness’ (p. 171) became the most quoted sentence from the book. Mantel’s account of those terrible days is masterly. But I suspect a further reason why endometriosis came to dominate the reviews was that readers felt competent to judge what had actually gone wrong on that occasion in a way that they did not when considering the events in the Secret Garden or on Blackpool Pier. The injustice with which Mantel was treated by her psychiatrist allowed them to disown the much more elliptical and diffuse aspects of Mantel’s narrative. It has to be conceded that at certain points in the memoir, Mantel seems to encourage this approach. She suggests, for instance, that the pains she complained of as a girl were early pathognomonic signs of what would develop into endometriosis (p. 187). The memoir exhibits a strange and surprising openness to biomedical explanation from this point on. But, as Sara L. Knox has rightly pointed out, nowhere does Mantel recant the supernatural account of her infirmities offered in the previous chapter.^32^

The idea that Mantel had a concurrent psychiatric condition was not far-fetched. She concedes as much herself. ‘I was labouring under a violent sense of injustice that may have seemed unreasonable to the people around me; I was angry, tearful and despairing, and I still had pains in my legs’ (p. 170). There was, moreover, an important relational context surrounding the onset of Mantel’s pains which, so far as I am aware, was not picked up by any of the reviewers. In 1972, Mantel decided to get married. Jack and her mother disapproved of her marriage and refused to sign her grant forms in protest. The mysterious pain, which she could not explain, occurred only *after* the rupture with Jack and her mother. Mantel appears to have experienced her undiagnosed illness as a repetition of some of the most traumatic aspects of her sufferings as a child. At the most basic level, endometriosis revived and amplified the colics and cramps and fevers that afflicted her from the age of five until she was well into her teens (p. 134). Its onset coincided with a collapse in her relationship with her mother and Jack, just as the non-specific illnesses of her childhood began when the conflicts between her mother and the rest of the family broke out into the open. Dr G’s response to her pains invited comparison with that of the GP who called her Little Miss Neverwell. It was bad enough that he was unwilling to explore a physical origin for her complaint. In claiming that her pains were hysterical – that they had been caused by a refusal to take her mother as her model and to put herself in what he plainly saw as a male gender role (that of a lawyer) – he was ignoring the highly charged meanings that male and female roles had had for Mantel throughout her childhood.

The relational context also matters because it shaped Mantel’s experience of her pains. It can be seen to inflect all the symptoms Mantel attributes to psychiatric medications. Consider this description of her response to Valium.

Valium, however, did work; it worked to damage me … One day I sat by the hearth at Roebuck Road and imagined myself starting fires – not in my own chimney, but fires in the houses of strangers, fires in the streets. Somewhere along the line, I seemed to have been damaged; I imagined myself doing damage, in my turn. I knew these thoughts were not rational, but I was obliged to entertain them; day by day I smouldered in a sullen fury, and when I saw a carving knife I looked at it with a new interest. I agreed to the clinic because I thought that, if I were to act on my impulses, someone would see me and stop me – before, at least, it got to arson and stabbing, and the deaths of strangers who had never harmed me at all. (p. 172)

Two of Mantel’s female relatives had died in fires: her paternal grandmother (who, significantly given the conflict generated by Mantel’s marriage, called off her wedding because she saw a ghost), and a relative of her mother, ‘a little girl called Olive who burned to death when her nightdress caught alight’ (p. 26). Perhaps Mantel’s wish to start fires was a way of expressing her feeling that, like Olive and her grandmother, she was being consigned to her family’s damaged past. In Mantel’s first two novels, Muriel Axon, a character Mantel has said she based on herself, stabs herself with a pair of scissors and sets fire to the house she grew up in, which is full of ghosts (the house is plainly modelled on Brosscroft).^33^

Many critics have commented eloquently on Mantel’s first attempt to write herself back to health during her stay on the psychiatric ward.^34^ This took the form of a short story. Here is how she explained it to Dr G.

And what was it about? A woman who believes her baby has been taken away and a substitute provided in its place. I see, said Dr G, and where and when did this occur? In rural Wales, I said, funnily enough. (I’d never been to Wales.) I don’t have to say the date but it feels like the early 192os. I mean judging by their furniture and clothes. Does it? said Dr G. It’s a time well before social insurance, anyway, I said. The doctor won’t come up the mountain to see them because they can’t pay. I see, said Dr G. And how does it end? Oh, badly. (pp. 173–4)

The next time Mantel saw Dr G, he forbade her to write. But what are we to make of this story? I suggest it is an attempt to see her mother from her grandmother’s point of view. Mantel’s mother was born in December 1926, a little after ‘the early 1920s’ but close enough.^35^ We know too that she saw herself as a changeling: ‘Cinderella was her favourite part. Her favourite scene: the Transformation. She asked herself, could she really be the child of her parents? Or some changeling princess, dropped into Bankbottom by accident?’ (p. 50). The Welsh setting too is significant. Mantel recalls that when she was growing up,

All my behaviour seemed to anger [Jack], just by the fact of *being* behaviour … I felt as if I were a survival, a relic, a small squat subject race, whose aboriginal culture was derided; like the Welsh, for example, a nation for whom Jack had no time at all. (pp. 143–4)

Wales here is surely a metaphor for Mantel’s grandparents’ household. From Jack’s standpoint, it was a contemptible, aboriginal place.

Mantel has said that the story she wrote during her psychiatric admission supplied the nucleus of her first two published novels, *Every Day is Mother’s Day* (1985) and *Vacant Possession* (1986). It is striking though that those novels are about three generations. In *Every Day is Mother’s Day*, Muriel Axon, a ‘mildly retarded’ young woman kills her child, whom her mother says is a changeling, to prevent her mother from taking it away from her. The reason no doctor comes to see her during her pregnancy is that her mother will not allow it. Her mother wants to control the pregnancy herself. Her chief rival is the state as represented by the local authority’s department of Social Services. (Muriel Axon goes on to kill her mother.) When Mantel first presented at the Student Health Centre, she and her doctors wondered if she was pregnant. Throughout the chapter dealing with her endometriosis, there are hints that an important but unacknowledged function of psychiatry and gynaecology is to demonstrate that the female body and everything it is capable of can be controlled by overwhelming force. Changelings represent the ultimate proof of this state of affairs: a mother whose child is taken away from her is deprived of the creativity of her own body. In an interview given in 1997, Mantel commented that

It’s a strange thing to say, considering that Muriel is a mentally defective murderess, but I really think that Muriel is me in that relationship, who can only cope by closing her eyes, closing her ears and … I think that if I go back to my childhood, probably the relationship between myself and my mother was negotiated very badly … it was a very long and painful process for me to see what that book was about. And once I got to the end of *Every Day is Mother’s Day* Muriel had in effect murdered a child to stop it being taken by her mother … Muriel has never gained a sense of her own personality, she has no self, because her mother has not allowed her to have a self.^36^

Perhaps some similar deprivation was the chief cause of Mantel’s anger when she was a young woman.

In a lengthy critique of the Paris School of Psychosomatics, the late André Green argued that some people are compelled to live out their conflicts through their bodies because of excessive mentalisation (the Paris School focused on cases of insufficient mentalisation).^37^ Such people are often required by parents to deny reality. Green suggested that this leads to a shrinkage of the preconscious mind, the mind they can go back to, to turn things over at their own pace. The distinction between internal and external reality is harder to maintain as a result. It seems to me unquestionable that Mantel was forced to live out a great deal of her emotional life through her body and that, because of the reign of secrecy at home concerning her parents’ living arrangements, she was required to deny reality both in the home and outside it, more or less constantly. Green says it is impossible to understand psychosomatic structures without recourse to the concept of ‘negative hallucination’ (denial of reality). Mantel’s tragedy is that the psychosomatic structure of her early home life robbed her of a standard of normality to apply to her pains. The pain she was left with long after leaving the psychiatric ward became the heir to countless childhood bafflements. It reached back to the childhood growing pains that her GP thought were psychosomatic; to the collapse of her family relationships at the age of seven (never to be discussed in public at all or in private very much); and in the psychiatric phase of her illness career, to the neurotic fears she found so real in early childhood. Her physicians’ denial that she had a physical illness can only have resonated with her early experience of having to be silent about her home life, a reticence that covered over so much loss for her.

In a fine essay on chronic pain and autobiography, Leigh Gilmore observes that ‘pain shapes the relationships we have to our bodies and with others’.^38^*Giving Up the* Ghost, which Gilmore singles out for special praise, illustrates this point with peculiar virtuosity. If we survey Mantel’s illness career up to her first hospital admission, we find that pain occupies the place previously taken by confidence about how matters stood within her family of origin. Pain is a means of retaining some of the *status quo ante*, albeit on worse terms. Of course, once she diagnosed the cause of her pains, she was able to separate them from her family life. But she did not always choose to do so. Early in the memoir, she describes visits that she and her husband used to make to her mother and Jack in Norfolk in the early 1990s. Jack, we are told, had recently had a coronary bypass. ‘Routinely, as we left’, Mantel writes, ‘there was a small ache behind my ribs’ (p. 18). Pain it seems still retained an object-relational significance.

Her physicians’ failure to diagnose the cause meant she had to carry it around the same way she carried around the memory of the evil spirit and the fizzing of the fly on the staircase: as a frightening somatic experience, expanding into something psychologically vast. So ingrained is this way of dealing with the vicissitudes of life that medical language has almost no purchase over it. Mantel cannot say that it *isn’t* her stepfather’s ghost she sees in her house in Norfolk because migraine auras were something she learned about long after her psychosomatic habits had been settled. She *has* to credit ghosts with a modicum of reality because ghosts are part of her own habitual mode of dialogue with herself, a mode that took shape in early childhood. Without them, she would not be who she is.

Medicine today finds itself in a peculiar position. Evidence from studies in epigenetics, epidemiology and infant research points to the power of childhood psychological experience in determining adult health. At the same time, medicine as it is practiced in hospitals, though officially wedded to a form of mind–body monism, in practice assigns little significance to mental functioning, which is always reduced to biology. Without denying the successes of biomedicine, books like Mantel’s show why interiority is so important. Interiority, understood diachronically and from a developmental point of view, plays a powerful role in shaping illness experience and can also decisively affect the course of a disease process. I ended my previous paper by suggesting that first-person memoirs offer theorists of life writing and others the opportunity to challenge the anti-mental bias of modern biomedicine by reclaiming the whole experiential field in which illness occurs in the West today. I hope I have demonstrated that Mantel’s memoir makes a vital contribution to that task.

## Notes

1. Hilary Mantel, *Giving Up the Ghost* (London: Fourth Estate, 2003). All page references to this book appear inline.

2. Couser is credited with this coinage in Sidonie Smith and Julia Watson's, *Reading Autobiography* (Minneapolis: University of Minnesota Press), p. 143. He first used it in correspondence with these authors.

3. Eric J. Cassell, *The Healer's Art: A New Approach to the Doctor-Patient Relationship* (Philadelphia: J. B. Lippincott, 1976), pp. 30–4.

4. Paul John Eakin, *Living Autobiographically: How We Create Identity in Narrative* (Ithaca, NY: Cornell University Press, 2008), especially chapter 2, pp. 61–86.

5. For an introduction to contemporary psychoanalytic psychosomatics, the reader is referred to Marilia Aisenstein and Elsa Rappaport de Aisemberg (eds.), *Psychosomatics Today: A Psychoanalytic Perspective* (London: Karnac and the International Psychoanalytic Association, 2010); Richard M. Gottlieb, ‘Psychosomatic Medicine: The Divergent Legacies of Freud and Janet’, *Journal of the American Psychoanalytic Association*, 51 (2003), pp. 857–81 and Catalina Bronstein, ‘On Psychosomatics: The Search for Meaning’, *International Journal of Psycho-Analysis*, 92 (2011), pp. 173–95.

6. Winnicott first used this term in a review of Carl Jung's autobiography. D.W. Winnicott, ‘Memories, Dreams, Reflections: By C. G. Jung’, *International Journal of Psycho-Analysis*, 45 (1964), pp. 450–5.

7. For this Kleinian inflection of Kristeva's distinction between the somatic and the symbolic, I am indebted to Catalina Bronstein. See her ‘Psychosomatics: the role of unconscious phantasy’ in Aisenstein and de Aisemberg, *Psychosomatics Today*, pp. 63–76 and especially ‘“I Am Not Crying. I Am Rubbing My Eyes”: Annie and the Hollow Object’, *Journal of Child Psychotherapy*, 40.2 (2014), pp. 135–49.

8. Two massive on-going studies on the links between early life stress and adult health outcomes are the Dunedin Study and the Adverse Childhood Experiences (ACE) Study at the US National Center for Disease Control in San Diego. The Dunedin Study is a long-term cohort study of 1037 people born between 1 April 1972 and 31 March 1973 in a maternity unit in Dunedin, New Zealand (http://dunedinstudy.otago.ac.nz). It is, by general consent, the most important study of its kind to date. The ACE Study (http://www.acestudy.org) is larger but is not double blind and lacked a prospective design at its outset.

9. The medical-anthropological literature in this area is vast. Two landmark studies are: Arthur Kleinman and Joan Kleinman, ‘How Bodies Remember: Social Memory and Bodily Experience of Criticism, Resistance, and Delegitimation following China's Cultural Revolution’, *New Literary History*, 25.3 (1994), pp. 707–23 and Andrew J. Strathern, *Body Thoughts* (Ann Arbor: University of Michigan Press, 1996). For examples of how narrative-based medicine approaches this area, see Brian Hurwitz, Trisha Greenhalgh and Vieda Skultans (eds.), *Narrative Research in Health and Illness* (Oxford: BMJ Books, 2004).

10. I will use the name ‘Hilary’ to designate Mantel's childhood self, reserving the name ‘Mantel’ for the adult.

11. This experience may underpin Mantel's Cromwell trilogy, currently in progress. Cromwell attaches himself to Top Monk, Wolsey, and flourishes under his tutelage.

12. Julia Kristeva, *Revolution in Poetic Language*, trans. Margaret Waller (New York: Columbia University Press) p. 47.

13. Hanna Segal, ‘Notes on Symbol Formation’, *International Journal of Psycho-Analysis*, 38 (1957), pp. 391–7; reprinted in extended form in Elizabeth Bott Spillius (ed.), *Melanie Klein Today: Developments in Theory and Practice* (London: Routledge and the Institute of Psychoanalysis, 1988, repr. 2003), Vol 1, pp. 160–77.

14. For the theory of memories in feeling, Melanie Klein, ‘Envy and Gratitude’ (1957)’, in Masud Khan (ed.), *Envy and Gratitude and Other Works 1946–1963* (London: The Hogarth Press and the Institute of Psycho-Analysis, 1964, repr. 1975), p. 104.

15. Samuel Taylor Coleridge (ed.) and Barbara Rooke, *The Friend*, 2 vols (London: Princeton UP, 1966) i, p. 419.

16. Hilary Mantel, ‘Diary’, *London Review of Books*, 25.20 (2003), pp. 30–1.

17. I have argued elsewhere that the metaphors used in Blackpool recur in her account of akathisia: see Neil Vickers, ‘Hilary Mantel and the Space of Life Writing’, in Eveline Kilian and Hope Wolf (eds.), *Life Writing and Space* (Farnham: Ashgate, 2016), pp. 57–71 (p. 69).

18. Vittorio Gallese and Corrado Sinigaglia, ‘What is So Special about Embodied Simulation?’, *Trends in Cognitive Sciences*, 15.11 (2011), pp. 512–9.

19. The issue is organised around a ‘target article’ by Leonhard Schilbach, Bert Timmermans, Vasudevi Reddy, Alan Costall, Gary Bente, Tobias Schlicht and Kai Vogeley, ‘Towards a Second-Person Neuroscience’, 36.4 (2013), pp. 393–414. The article is followed by 28 papers setting out responses by neuroscientists and others.

20. Daniel N. Stern, *The First Relationship: Infant and Mother* (Cambridge, MA: Harvard University Press, 2002), pp. 42–3; G. Kugiumutzakis, T. Kokkinaki, M. Markodimitraki and E. Vitalaki, ‘Emotions in Early Mimesis’, in Jacqueline Nadel and David Muir (eds.), *Emotional Development: Recent Research Advances* (Oxford: Oxford University Press, 2005), pp. 161–82.

21. Maurice Merleau-Ponty, ‘The Child's Relations With Others’, in James M. Edie (ed.) *The Primacy of Perception* (Evanston, IL: Northwestern University Press, 1964), pp. 96–155 (p. 118). Merleau-Ponty credits Husserl with these ideas in that paper.

22. This point has also been made by Giuseppe Civitarese in ‘Between “Other” and “Other”: Merleau-Ponty as a Precursor of the Analytic Field’, *fort da*, 20.1 (2014), pp. 9–29.

23. For an excellent overview of the medical literature, see the papers in Ruth A. Lanius, Eric Vermetten and Clare Pain (eds.), *The Impact of Early Life Trauma on Health and Disease* (Cambridge: Cambridge University Press, 2010).

24. ‘Femininity, Sexuality and Inner Space’ in Henri Rey (ed.), *Universals of Psychoanalysis in the Treatment of Psychotic and Borderline States* (London: Free Association Books, 1994), pp. 263–76.

25. ‘My mother lights up with indignation when she speaks of the new flats, and her incandescent hair glows around her head. “It's scandalous! It's ridiculous! They’re moving them in before the light fittings have been put up! No curtain rails between the lot of them.” I take Evelyn down to the end of the yard. I lead her in a game, called “Talking about the New Flats”. We put our hands on our hips. We stare furiously over the wall. We shout: “It's scandalous. It's ridiculous!”’ (p. 71).

26. Soon after Jack appears in the book, we get this:
I am looking for someone to marry. It's a business I want to get settled up. I hope Jack might do, though it is a pity he is not my relative. He is just someone we know. I am, I already know, the kind of person who would marry back into my own family (pp. 82–3).

27. Ian was born in June 1957, when Hilary was nearly five. If she was seven when this scene occurred, there is an 11 in 12 chance he was 2 years of age. http://www.freebmd.org.uk/cgi/information.pl?r=155333695:8377&d=bmd_1448300351.

28. Note also this:
I thought I should never be free of Malachy, never reach the age of eleven; I felt that she had absorbed me, drawn me to her by my woolly cardigan, by means of static electricity: drawn me into the fusty depth of her habit, and then leached me into her thick body, as if she were my mother indeed, and I was beating at the walls of her nunly womb (p. 128) No male is credited with comparable powers.

29. Hilary Mantel, ‘How Much Pain is Too Much Pain?’, *International Association for the Study of Pain Insight* (2013), pp. 8–12 (p. 10).

30. Alan Radley, *Works of Illness: Narrative, Picturing and the Social Response to Serious Disease* (Ashby-de-la-Zouch: Inkerman Press, 2009), p. 136. A similar line is taken by Inga Clendinnen, ‘The Crack in the Teacup: Reading Hilary Mantel’, *Australian Book Review*, 256 (2003), pp. 35–40, though the latter sees them as neurological auras.

31. Eileen J. Pollard, ‘Mind What Gap?’: An Interview with Hilary Mantel’, *Textual Practice*, 29.6 (2015), pp. 1035–44 (p. 1042).

32. Sara L. Knox, ‘Giving Flesh to the “Wraiths of Violence”: Super-Fiction in the Writings of Hilary Mantel’, *Australian Feminist Studies*, 25.65 (2010), pp. 313–23 (p. 319).

33. Muriel stabs herself with scissors in *Every Day is Mother's Day* (London: Chatto & Windus, 1985), p. 29. Her arson attack on the home she grew up in on p. 10 of *Vacant Possession* (London: Chatto & Windus, 1986).

34. See for instance Amy Prodromou, ‘“That Weeping Constellation”: Navigating Loss in Memoirs of Textured Recovery’, *Life Writing*, 9.1, pp. 57–75 (p. 71); Robert Kusek, ‘“To Seize the Copyright in Myself.” *Giving Up the Ghost* by Hilary Mantel as an Exercise in Autopathography’, *Studia Literaria*, 24.3 (2014), pp. 177–90; Eileen J. Pollard, ‘“But at Second Sight, the Words Seemed Not So Simple” (Woolf, 1929): Thickening and Rotting Hysteria in the Writing of Hilary Mantel and Virginia Woolf’, *Virginia Woolf Miscellany*, 80 (Fall, 2011), pp. 24–6 (p. 25); Joseph Brooker, ‘Around 2000: Memoir as Literature’, in Adam Smyth (ed.), *A History of English Autobiography* (Cambridge: Cambridge University Press, 2016), pp. 374–87 (p. 382); Patricia Waugh, ‘Memory and Voices: Challenging Psychiatric Diagnosis through the Novel’, in Sebastian Groes (ed.), *Memory in the Twenty-First Century: New Critical Perspectives from the Arts, Humanities, and Sciences* (Houndmills: Palgrave, 2016), pp. 316–24.

35. http://www.freebmd.org.uk/cgi/information.pl?r=155333695:8377&d=bmd_1448300351.

36. Rosario Arias, ‘An Interview with Hilary Mantel’, *Atlantis*, 20.2 (1998), pp. 277–89.

37. André Green, ‘Thoughts on the Paris School of Psychosomatics’, in Aisenstein and de Aisemberg, *Psychosomatics Today*, pp. 1–47.

38. Leigh Gilmore, ‘Agency Without Mastery: Chronic Pain and Posthuman Life Writing’, *Biography*, 35.1 (2012), pp. 83–98 (p. 84).

